# Understanding Psychosocial and High-Risk Sexual Behaviors Among Detained Juveniles: A Descriptive Study Protocol

**DOI:** 10.2196/resprot.5153

**Published:** 2015-12-30

**Authors:** Madison L Gates, Michelle Staples-Horne, Jeanne Cartier, Candace Best, Veronica Walker, David Schwartz, Wonsuk Yoo

**Affiliations:** ^1^ Institute of Public and Preventive Health Department of Family Medicine, Medical College of Georgia Georgia Regents University Augusta, GA United States; ^2^ Georgia Department of Juvenile Justice Atlanta, GA United States; ^3^ College of Nursing and Allied Health Professions University of Louisiana Lafayette Lafayette, LA United States; ^4^ Department of Psychological Sciences Georgia Regents University Augusta, GA United States; ^5^ Lexington Public Library Lexington, KY United States; ^6^ Institute of Public and Preventive Health Georgia Regents University Augusta, GA United States

**Keywords:** adolescent, African Americans, HIV infections, mental health, risk factors, risk reduction behavior, sexual behavior, sexually transmitted diseases, spouse abuse, unsafe sex

## Abstract

**Background:**

African American women are disproportionately impacted by sexually transmitted infections (STIs), such as chlamydia and gonorrhea, which are known risk factors for human immunodeficiency virus (HIV) infection. STIs, particularly chlamydia and gonorrhea, are even more prevalent among young African American women with a juvenile detention history. The population with experiences with the criminal justice system has greater rates of STIs and is diagnosed more often with mental health issues, often related to sexual abuse or intimate partner violence, compared to peers who have not been detained by law enforcement. Psychosocial factors, especially those related to intimate relationships (ie, the imperativeness of being in a relationship and the power one has in their relationship), have emerged as important explanatory factors for acquiring STIs, including HIV, and a component of risk reduction interventions.

**Objective:**

To investigate more comprehensively the relationship between psychosocial risk factors and STIs, including HIV, as it relates to reduction and prevention of these diseases. The long-term goal is to improve the effectiveness of evidence-based interventions with a major focus on intimate relationship dynamics.

**Methods:**

This descriptive study surveys young women (ages 13-17) who have been detained (incarcerated) by a department of juvenile justice. In addition to being female and detained, eligibility criteria include being detained longer than 30 days and being free of cognitive impairments. This study will include young women from one juvenile detention center. The primary outcomes to be measured are STI knowledge, intimate relationship dynamics (ie, imperativeness and power), and high-risk sexual behaviors. High-risk sexual behaviors will be assessed using data extracted from health records.

**Results:**

Preliminarily, we have received assent from 26 primarily young African American women. The majority of participants (81%) had inadequate knowledge about STIs, 52% perceived a lack of power in their relationship, 56% were fearful of negotiating condom use, and 60% were not comfortable refusing sex. Interestingly, a majority of participants (68%) did not perceive a relationship as imperative.

**Conclusions:**

When enrollment and data collection are completed, it is expected that the primary outcome of intimate relationship dynamics (ie, imperativeness and power) will be associated with high-risk sexual behaviors and having an STI. Further, the findings are expected to provide guidance in developing a risk reduction intervention, for the population in which psychosocial factors related to intimate relationships will be central.

## Introduction

The National HIV/AIDS Strategy for the United States has made risk reduction, prevention, and treatment a high priority for populations most affected by and those at great risk for acquiring the disease [1]. African American women represent 63.5% of new human immunodeficiency virus (HIV) infections across all racial and ethnic female populations, with the rate of infection for this group estimated to be 228.8 per 100,000; this is among the highest in the southeast [2-5]. Further, African American women are disproportionately impacted by sexually transmitted infections (STIs), such as chlamydia and gonorrhea, which are known risk factors for HIV infection [6-10].

Understanding the relationship between HIV and other STIs, such as chlamydia, gonorrhea, and herpes simplex virus (HSV), is a component of the National HIV/AIDS Strategy for the United States [1], particularly with respect to the effort to reduce and prevent the disease. This study aims to investigate more comprehensively the relationship between psychosocial risk factors and STIs, including HIV, as it relates to reduction and prevention of these diseases. STIs are a significant health issue among young adults (≥ 20 years old) in Georgia and across the nation [6,11,12]. In 2014, the Centers for Disease Control and Prevention (CDC) reported that STIs were particularly problematic in Georgia and in the south in general [6]. Georgia ranked ninth in the United States for chlamydia, eighth for gonorrhea, and first for primary and secondary syphilis [6].

African American females (adolescents and adults) are at greater risk of acquiring chlamydia and gonorrhea than any other racial or ethnic female population [2,13-17]. STIs, particularly chlamydia and gonorrhea, are even more prevalent among young African American women with a juvenile detention history [10]. Despite the disproportionate rate of STIs among young African American women with a juvenile detention history and their risk for HIV, only a few studies related to these diseases have included this population [10]. We examined a similar population (young African American women who are vulnerable but not detained) to understand risk factors related to STIs/HIV and our population.

Vulnerable young African American women without a history of incarceration share similar characteristics with young African American women with a juvenile detention history, such as limited educational achievement, low socioeconomic status, limited utilization of health care services, unprotected sex, and substance use [18,19]. Studies of vulnerable young African American women suggest that these individuals exhibit high-risk sexual behaviors compared to nonvulnerable peers, have more lifetime sexual partners, engage in sex while high on drugs and alcohol, have low condom self-efficacy, do not use condoms consistently, and are more likely to have STIs [15,18,20].

Despite similarities between young African American women with and without juvenile detention history, the population with criminal justice system experiences has a greater rate of STIs and is diagnosed more often with mental health issues, often related to sexual abuse or intimate partner violence (IPV), compared to peers who have not been detained by law enforcement [10,18,19]. Studies investigating trauma (ie, sexual abuse and IPV) among populations who do not have a detention history have found a strong association between psychosocial factors (eg, intimate relationship dynamics), incidence of STIs, and risk of HIV [16,17,21].

Psychosocial factors, especially those related to intimate relationships (ie, the imperativeness of being in a relationship and the power one has in a relationship), have emerged as an important explanatory factor in the acquisition of STIs, including HIV, and component of risk reduction interventions [16,17,22-24]. As psychosocial and explanatory factors for STI/HIV risk, intimate relationships require much further investigation, given the history of physical abuse, sexual exploitation, and mental health issues experienced by many young women, and particularly young African American women, with a juvenile detention history.

## Methods

### Design

This descriptive study surveys young women (ages 13-17) who have been detained (incarcerated) by a department of juvenile justice (DJJ). This study aims to understand the association between STIs/HIV and psychosocial factors (intimate relationship dynamics) in a juvenile justice population, which has higher rates of mental health issues, substance use disorders, and trauma (eg, sexual abuse and exposure to violence) than the population without a criminal justice history. Further, this study aims to identify explanatory risk factors related to STIs. The long-term goal is to improve the effectiveness of evidence-based interventions (EBIs) that have a major focus on intimate relationship dynamics.

### Participants

This study will include young women from one juvenile detention center. The DJJ where the study will take place has three primary levels of supervision: community (probation and parole), regional youth detention centers (short-term detention), and youth development campuses (long-term commitment). The population for this study will come from a regional youth detention center (RYDC). We identified the population at the RYDC as appropriate for our investigation into STIs and psychosocial factors, since this population likely will have had more recent experiences outside detainment and psychosocial issues (eg, intimate relationships) may be more salient compared to peers residing at a youth developmental campus (YDC).

Young women projected to have commitments less than 30 days will be excluded, since stays of this duration likely will not allow sufficient time to recruit parents/guardians via mail and during on-site visitations, which occur two days per week (Tuesdays and Saturdays). Further, the population undergoes physical, mental/behavioral, dental, and security assessments upon entry into a facility; these assessments require several days, and during this time study personnel will not have access to the population. The inclusion criteria are listed in Table 1.

**Table 1:** Inclusion and exclusion criteria.

Characteristic/Factor	Inclusion	Exclusion
Facility	Regional Youth Detention Center	All other facilities
Race	All	None
Gender	Female	Male
Age	≥ 13	≤ 12
Projected detention	≥ 30 days	< 30 days
Cognitive capacity	Nonimpaired	Impaired
Reoffense	Allowable	Not applicable

### Recruitment

Investigators will collaborate with facility staff to identify young women who meet the inclusion requirements. Once a list of eligible participants is created, parents/guardians of minors or nonemancipated youths will be recruited via mail and during on-site visitations.

After recruitment of parents/guardians, investigators will recruit adolescents. Participants who meet the inclusion criteria will be recruited for one session; interactions with the young women will require approximately 15 minutes. In addition to being detention centers, RYDCs function as schools, providing a similar structure that would be found in a school district outside of the juvenile justice system. Thus, recruitment will occur weekly at the end of a health education class, where potential participants will be invited to participate in a brief paper survey that will require approximately 5 minutes to complete. The survey will be administered face-to-face by study personnel during the health education class.

Attrition will be measured using an enrollment log that will list participants who assent/refuse to participate, parents/guardians who consent, youths who withdraw their assent, and the population that is released earlier than anticipated. The enrollment log will allow study personnel to identify potential reasons why participants were lost from the study (eg, early release).

### Assent and Informed Consent

All participants will be required to sign an IRB approved assent form (≤ 17 years old) or consent form (18 and older). Consent of parents/guardians will be required for youths 17 years old and younger. Parents/guardians will be mailed a consent form with a self-addressed and stamped envelope to return their document to the lead investigator. Investigators will follow up in one week with parents/guardians who have not responded. Consent of parents/guardians also will be sought during visitations with their child/children.

Once parental/guardian consent has been obtained, assent will be sought from potential participants who are minors. To minimize embarrassment for adolescents whose parents/guardians do not consent to their participation and to limit peer pressure, all participants will be provided a research packet (two assent/consent forms and one survey) during a health education class. Participants will retain a copy of the assent/consent form and return one to the investigator. Investigators will explain to potential participants what the study entails, what is expected to be learned, why it is important, what participants’ contribution will be, their right to participate or not, and the fact that participation will have no impact on parole or leniency, as mandated by the Code of Federal Regulation, U.S. Department of Health & Human Services, Protection of Human Subjects (45 CFR 46) [25].

### Hypotheses

This study hypothesizes that young women (13 and older) who have low STI knowledge and relationship power, but high relationship need, will have greater odds of reporting high-risk sexual behaviors, having a mental or behavioral health issue, and being diagnosed or treated for at least one STI (Hypothesis 1). We also hypothesize that there will be race and ethnic disparities for STIs (Hypothesis 2).

### Outcome Measures

The primary outcomes to be measured are knowledge, intimate relationship dynamics (ie, imperativeness and power), and high-risk sexual behaviors. Knowledge about STIs will be measured using the STD-Knowledge Questionnaire (STD-KQ), a 27-item instrument with a Cronbach alpha of .86 and test-retest reliability of .88 [26]. Intimate relationship dynamics will be measured using an instrument developed at Emory University’s Center for AIDS Research. The instrument is comprised of 27 items, which assess relationship imperativeness and three psychosocial factors (ie, relationship power, self-efficacy to refuse sex, and fear of abuse) [21]. The subscales of relationship power, self-efficacy to refuse sex, and fear of abuse have Cronbach alphas of .70, .87, and .89, respectively [21]. High-risk sexual behaviors will be assessed using the following data from participants’ health records: history of substance use, history as a commercial sex worker, placement in foster care, homelessness, history of having a mental/behavioral health diagnosis, and history of having more than one sexual partner at a time.

### Planned Analyses

Statistical analyses will be conducted using SAS 9.4. Frequencies and proportions will be used to describe all discrete data. Means, medians, and standard deviations will be used to describe continuous data. Logistical regression models will be used to identify significant psychosocial factors (relationship imperativeness, relationship power, perceived self-efficacy to refuse sex, and fear of abuse), high-risk sexual behaviors, STI knowledge, and demographics (eg, race, ethnicity, and age). Odds ratios and 95% confidence intervals also will be reported. The chi-square (χ^2^) statistic or Fisher’s exact test will be employed to determine whether or not the rate of STIs are different across populations.

### Ethics

This protocol has been approved by the Institutional Review Board at Georgia Regents University (protocol number 631921-4) and by the Research Review Committee of the Georgia Department of Juvenile Justice.

## Results

This study was launched in February 2015 and is actively recruiting participants. Preliminarily, 26 out of 37 young women assented to participate, reflecting a 70% assent rate. We have not asked participants to give reasons for their refusal to participate, since the survey is administered in a classroom setting, and sharing these reasons may result in participant discomfort or their perception of the question as a form of coercion. Most participants are African American and their mean age is 15.75 years (SD 1.22) (see Table 2).

**Table 2:** Preliminary demographics.

	Age					
	13	14	15	16	17	Total
	n (%)					
African American	2 (8)	2 (8)	2 (8)	5 (21)	5 (21)	16 (67)
Hispanic/Latina	0 (0)	0 (0)	3 (13)	1 (4)	0 (0)	4 (17)
White	0 (0)	0 (0)	2 (8)	1 (4)	1 (4)	4 (17)
Total	2 (8)	2 (8)	7 (29)	7 (29)	6 (25)	24^a^

^a^ Missing data = 2

Preliminary data for the knowledge outcome indicate that 81% (21/26) of participants responded incorrectly to at least 7 out of 12 items regarding STIs. A median split, as used by Raiford, Seth, and DiClemente in a related study [21], defined high and low responses for power, fear of abuse, and sex refusal. A majority of participants, 52% (13/25), perceived they did not have power in their relationships, 56% (14/25) feared negotiating condom use with partners, and 60% (15/25) perceived a lack of self-efficacy to refuse sex. However, emerging data also indicate that 68% (17/25) of current participants did not perceive relationships as imperative.

## Discussion

### Preliminary Findings

The primary outcome of intimate relationship dynamics (ie, imperativeness and power) is expected to be associated with high-risk sexual behaviors and having an STI, as found in populations without a history of juvenile detention in related studies. A study that included young African American women (ages 15-21), who were primarily inner-city youths seeking sexual health services from a community agency, found that participants who perceived their relationships as imperative (1) had less relationship power, (2) were more likely to perceive themselves as being unable to refuse sex, (3) were more likely to fear negotiating condom use with their partners, and (4) were more susceptible to partner abuse [21]. Furthermore, participants who perceived relationships to be imperative reported having sex while under the influence of alcohol and drugs and being willing to engage in unprotected sex [21].

Paxton et al, in a study that included African American women (ages 26-54), found that participants stayed with unfaithful partners largely due to the desire to have a relationship [24]. African American women participating in the study reported high-risk sexual behaviors, such as knowingly having sex with partners who have STIs, not requiring unfaithful partners to use condoms, only requiring unfaithful partners to use condoms with other sexual partners, and engaging in sex while high on drugs and alcohol [24]. The work of Raiford, Seth, and DiClemente [21] and Paxton et al [24] suggests that intimate relationship dynamics (imperativeness and power) are salient factors for both adolescent and adult women.

### Limitations

The primary limitation is expected to be participant attrition. The population of interest for this study is housed in an RYDC, which means that it is highly fluid, resulting in recruitment challenges and a small sample size [27]. Many potential participants satisfied all inclusion criteria except for detainment length. Despite the high turnover of this population, psychosocial factors may be more salient for the RYDC group, who have more recent free-world experiences than detainees in YDCs.

The study design only included young women currently being detained and not the perspective of young men with a history of juvenile detention. The nature of intimate relationships between young women and men, and specifically factors related to high-risk sexual behaviors, have not been well defined. This study did not seek to contact intimate partners of participants because of many potential issues, such as revisiting traumatic experiences, partners being detained/incarcerated, or relationships violating consent laws. Furthermore, few studies have investigated the dyad relationship of young women and men in regard to imperativeness and power; the majority of the studies reviewed included adolescent and adult women.

Future investigations may address high turnover and attrition by extending studies into the community for participants who are being released or who have detention durations of less than 30 days. In other words, future studies may include populations with current or past experiences with the juvenile justice system. There also may be technological tools, such as mobile applications and social media, to facilitate outreach into the community for the juvenile justice population being released. Outreach beyond detention centers also may facilitate including dyads to understand relationship dynamics more completely from the perspectives of young women and men, which may lead to more effective interventions.

## Abbreviations

DJJ: department of juvenile justice
EBI: evidence-based intervention
IPV: intimate partner violence
RYDC: regional youth detention center
STI: sexually transmitted infection
YDC: youth developmental campus
